# Identification and modelling of fast and slow *I*_h_ current components in vestibular ganglion neurons

**DOI:** 10.1111/ejn.13021

**Published:** 2015-08-06

**Authors:** Christophe B. Michel, Christine Azevedo Coste, Gilles Desmadryl, Jean‐Luc Puel, Jerome Bourien, Bruce P. Graham

**Affiliations:** ^1^ Computing Science & Mathematics School of Natural Sciences University of Stirling Stirling FK9 4LA UK; ^2^ DEMAR Team INRIA/LIRMM University of Montpellier Montpellier Cedex 5 France; ^3^ Institut National de la Sante et de la Recherche Medicale Unite Mixte de Recherche 1051 Institut des Neurosciences de Montpellier Montpellier France; ^4^ Universite Montpellier 1 & 2 Montpellier France

**Keywords:** computational modelling, hyperpolarization‐activated cation current, vestibular, voltage clamp

## Abstract

Previous experimental data indicates the hyperpolarization‐activated cation (*I*_h_) current, in the inner ear, consists of two components [different hyperpolarization‐activated cyclic nucleotide‐gated (HCN) subunits] which are impossible to pharmacologically isolate. To confirm the presence of these two components in vestibular ganglion neurons we have applied a parameter identification algorithm which is able to discriminate the parameters of the two components from experimental data. Using simulated data we have shown that this algorithm is able to identify the parameters of two populations of non‐inactivated ionic channels more accurately than a classical method. Moreover, the algorithm was demonstrated to be insensitive to the key parameter variations. We then applied this algorithm to *I*_h_ current recordings from mouse vestibular ganglion neurons. The algorithm revealed the presence of a high‐voltage‐activated slow component and a low‐voltage‐activated fast component. Finally, the electrophysiological significance of these two *I*_h_ components was tested individually in computational vestibular ganglion neuron models (sustained and transient), in the control case and in the presence of cAMP, an intracellular cyclic nucleotide that modulates HCN channel activity. The results suggest that, first, the fast and slow components modulate differently the action potential excitability and the excitatory postsynaptic potentials in both sustained and transient vestibular neurons and, second, the fast and slow components, in the control case, provide different information about characteristics of the stimulation and this information is significantly modified after modulation by cAMP.

## Introduction

The identification and characterisation of neuronal ionic conductances remains a major component of electrophysiological research. Classically, individual ionic conductances are pharmacologically isolated, and their current responses are recorded by the voltage‐clamp technique. For each voltage step, the corresponding current response is fitted in order to identify its magnitude and time constant parameters (Hodgkin & Huxley, 1952). This method extracts these characteristics from each voltage‐clamp trace independently. Consequently we will call this parameter identification approach the single‐trace method. An improved method that simultaneously extracts the conductance characteristics using all voltage‐clamp traces at once has been shown to produce more accurate solutions in the identification of sodium currents (Willms *et al*., 1999). This algorithm is called the full‐trace method.

In some cases, pharmacological tools to isolate individual ionic currents are not available. This is the case for the hyperpolarisation‐activated cation currents in the cochlea (Chen, 1997; Yi *et al*., 2010). In the vestibular ganglion, the fitting of these currents was significantly better with a two‐component exponential model (Meredith *et al*., 2012), and biochemical analysis showed the presence of different HCN subunits in the vestibular periphery (Horwitz *et al*., 2010, 2011). These studies could not draw conclusions about the differences in activation of the two apparent components, due to the limited performance of the single‐fitting approach. Given its better constraints, we assumed that the full‐trace method should be a good tool for analysing the vestibular ganglion *I*_h_ currents, first, in order to corroborate the presence of two components and, second, to isolate analytically rather than pharmacologically these two components and so permit their individual modelling to test their electrophysiological significance.

We first validated on simulated data the ability of the full‐trace method to discriminate two populations of non‐inactivated voltage‐dependent ionic currents. Then, we applied the method to *I*_h_ currents recorded from mouse vestibular ganglion neurons and identified the activation and kinetics of both components. Finally, these two *I*_h_ components were tested individually in a computational auditory neuron model (Rothman & Manis, 2003a) modified to reproduce the regular and irregular (sustained and transient) vestibular ganglion neuron firing patterns (Kalluri *et al*., 2010; Yoshimoto *et al*., 2015), in the control case and in presence of cAMP, an intracellular cyclic nucleotide that modulates hyperpolarization‐activated cyclic nucleotide‐gated (HCN) channel activity (Chen, 1997; Yi *et al*., 2010). The results suggest that the fast and the slow components modulate differently action potential (AP) excitability and excitatory postsynaptic potentials (EPSPs) in the sustained and transient vestibular ganglion neurons. In addition, the fast component allows the neuron to fire a single AP at the end of a hyperpolarizing stimulation whatever its duration or amplitude, whereas the slow component, in the control case, allows the neuron to fire a number of APs correlated with both the stimulation duration and amplitude and, after modulation by cAMP, it results in a number of APs exclusively correlated to the stimulation amplitude.

## Materials and methods

### Patch‐clamp recordings

Whole‐cell recordings of hyperpolarisation‐activated currents were performed in postnatal day (P)5–P8, male and female, wild‐type Swiss mice (CERJ, Le Genest, France), specifically in the superior branch of the vestibular nerve, innervating the utricular macula and the horizontal and lateral cristae, using an isolation procedure previously described (Chabbert *et al*., 2001a). Ganglia were aseptically dissected from mice rapidly killed by decapitation (the day of birth was considered P0). About twenty ganglia for each experiment were collected in phosphate‐buffered saline (PBS; Life Technologies). We tested different incubation times and trypsin concentrations and found no difference in the amplitude or the shape of the recorded currents. We settled on protocols employing trypsin at 0.25% for 12 min at 37 °C in PBS containing 0.25% EDTA‐trypsin (Life Technologies). Ganglia were triturated with fire‐polished Pasteur pipettes of three decreasing diameters in a cell culture medium containing Neurobasal medium (Life Technologies), 10% B27 (Life Technologies), 25 μm glutamate and 0.25 mm glutamine. Neurons were plated onto 35‐mm culture dishes (Nunc) coated with 10 μg/mL poly‐D‐ornithine (Sigma) in cell culture medium. Cells were used between 1 and 4 h after dissociation. Under phase‐contrast microscopy, dissociated neurons had a spherical shape and birefringent cytoplasm as previously reported (Desmadryl *et al*. 1997). Cell diameters ranged between 10 and 25 μm in a sample of 20 animals. Only isolated neurons exhibiting no processes were chosen for the electrophysiological studies.

The standard extracellular solution contained (in mm): NaCl, 135; KCl, 5; HEPES, 10; glucose, 10; and MgCl_2_, 1. Patch pipettes (2 and 3 MΩ) were filled with the following intracellular solution (in mm): KCl, 135; EGTA, 10; HEPES, 25; MgATP, 3; NaGTP, 1; and glucose, 10. The pH of the solution was adjusted to 7.35 and the osmolarity to 300 mOsm/L. Data were recorded with an Axopatch 200B and analysed with Pclamp software (Axon Instrument, Foster City, CA, USA; now Molecular Devices, part of Danaher). Series resistances in the range 5–9 MΩ were 80% compensated after cancellation of the capacitive transients. Data presented are corrected online for junction potential (−7 mV). Delayed‐rectifier and low‐voltage‐activated potassium currents are not activated under −60 mV (Chabbert *et al*., 2001a), and KCNQ currents are not active at these ages at potentials under −70 mV (Hurley *et al*., 2006). *I*_h_ currents were isolated by subtracting traces evoked in the presence of 5 mm BaCl, known to abolish the instantaneous component, from those elicited in control external solution (Chabbert *et al*., 2001b). The care and use of animals followed the animal welfare guidelines of the Institut National de la Sante et de la Recherche Medicale (Inserm), and was approved by the Ministere Français de l'Agriculture et de la Peche (authorization number A 3417231).

### Current, activation and kinetic models

The form of current equation initially proposed by Hodgkin & Huxley (1952) in the context of sodium and potassium channels is adapted here for the hyperpolarisation‐activated cation current *I*_h_ to give:

(1) $$I_{h}\left(t\right)=G_{h}\left(r_{\infty }\left(V_{s}\right)+\left(r_{\infty }\left(V_{\mathrm{ps}}\right)-r_{\infty }\left(V_{s}\right)\right)\text{exp}\left(-t/\tau_{r}\left(V_{s}\right)\right)\right).\left(V_{s}-E_{h}\right)$$

where *G*_h_ is the maximal conductance, *r*_∞_ is the activation function, *τ*_*r*_ is the activation kinetic function, *V*_ps_ is the voltage pre‐step value, *V*_s_ is the voltage‐step value and *E*_h_ is the reversal potential. Param *τ*_*r*_ eter identification involves determining values for the parameters *G*_h_, *r*_∞_ and *τ*_*r*_ from recordings of *I*_h_. The reversal potential is predetermined and here is set to *E*_h_ = −36 mV to correspond to physiological values (Chen, 1997; Meredith *et al*., 2012).

Functional forms for *r*_∞_ and *τ*_*r*_ as functions of the membrane voltage V are assumed. The conductance activation *r*_∞_ as a function of voltage is commonly described by a Boltzmann function of the form:

(2) $$r_{\infty }\left(V\right)={\left(1+\text{exp}\left(-\left(V-V_{h}\right)/k\right)\right)}^{-1}$$

where *V*_h_ is the half‐activation voltage and *k* is the slope factor that determines the steepness of the Boltzmann function.

The conductance kinetic *τ*_*r*_ as a function of voltage can be described by a Gaussian function (Izhikevich, 2010) of the form:

(3) $$\tau_{r}\left(V\right)=B+A.\text{exp}\left(-{\left(M-V\right)}^{2}/S^{2}\right)$$

where *M*,* S* and *A* are respectively the location of the maximal value, the width (σ for a Gaussian) and the amplitude of the Gaussian, and *B* is an offset from the abscissa axis (base time constant).

These two curves (activation and kinetic) are called the characteristic curves of a voltage‐dependent channel population and, along with the maximal conductance *G*, they are sufficient to fully describe the resulting current.

### Algorithms

This study aims to show that the full‐trace method (Willms *et al*., 1999) is more reliable than the more widely used single‐trace approach for identifying the parameters of currents composed of two components with differing activations and kinetics. In other words, the recorded membrane currents are the sum of two conductances described by the above equations, so the parameter identification algorithms have to identify the parameters of currents described by the equation:

(4) $$I_{\mathrm{total}}=I_{\mathrm{hs}}\left(G_{s},r_{s\infty }\left(V\right),\tau_{\mathrm{rs}}\left(V\right)\right)+I_{\mathrm{hf}}\left(G_{f},r_{f\infty }\left(V\right),\tau_{\mathrm{rf}}\left(V\right)\right)$$

Here *I*_hs_ and *I*_hf_ are the slow and fast components of the total current and $r_{s\infty }\left(V\right)$, $\tau_{\mathrm{rs}}\left(V\right)$, $r_{f\infty }\left(V\right)$, $\tau_{\mathrm{rf}}\left(V\right)$ are their respective activation and kinetic functions.

#### The single‐trace method

**Figure 1:**
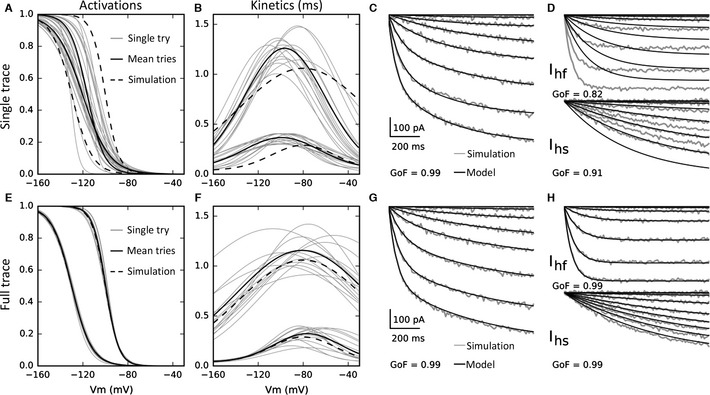
Single‐trace and full‐trace method identification and reconstructions. The single‐ and full‐trace method individual activation and kinetic identifications (single try) are shown in thin and the average in bold (mean of 14 best tries). (A) The activations of the two components identified by the single‐trace method are confounded and the averages quasi‐superposed. (B) The single method‐identified kinetics are well separated. (C) The single‐trace method permits a very accurate reconstruction of the two‐component combined current (GoF = 0.99), but (D) does not permit the accurate reconstruction of individual component currents (*I*_hf_ GoF =  0.82, *I*_hs_ GoF = 0.91). (E) The activations of the two components identified by the full‐trace method are very well separated, superposed on the original current curves (dashed lines). (F) The full‐trace method‐identified kinetics are well separated. (G) The full‐trace method permits a very accurate reconstruction of the two‐component combined current (GoF = 0.99), and (H) does permit the accurate reconstruction of the individual component currents (*I*_hf_ GoF = 0.99, *I*_hs_ GoF = 0.99).

A voltage‐clamp protocol consists of a pre‐step potential *V*_ps_ imposed on the cell followed by several values of a step potential *V*_s_, completed with a return to the pre‐step potential (deactivation). When *V*_ps_ is different from *V*_s_, a current flows through the membrane (activation) and is measured by the patch‐clamp setup. The traces of a voltage‐clamp experiment are the individual currents recorded in response to different voltage steps in the voltage‐clamp protocol. The single‐trace method used here fits each current trace (Fig. 1A) of a voltage‐clamp experiment with the current Eqn (1) and identifies, for each value of *V*_s_, the variables $r_{\infty }\left(V_{s}\right)$, $\tau_{r}\left(V_{s}\right)$, and *G* (*V*_s_) (Rothman & Manis, 2003b; Gerstner, 2014). To enable identification of these parameters, we set the initial value of activation to the steady‐state value at the pre‐step potential $r_{\infty }\left(V_{\mathrm{ps}}\right)$ (Willms *et al*., 1999), which is 0 for the *I*_h_ current, and a value for the ionic reversal potential is assumed. From the resulting sets of parameter values, first the maximal conductances are averaged to give a single estimate of the maximal conductance *G*. Second, the set of values for $r_{\infty }\left(V_{s}\right)$ are fitted with the activation Eqn (2), yielding values for the parameters *V*_h_ and *k*. Finally, the set of identified values for $\tau_{r}\left(V_{s}\right)$ are fitted with the kinetic Eqn (3) in order to evaluate the parameters *M*,* S*, A, and *B*.

When the current recorded during a voltage‐clamp experiment is the sum of two components, as it is here, the fitting has to identify twice the variables (one set for each component): $r_{s\infty }\left(V_{s}\right)$, $\tau_{\mathrm{rs}}\left(V_{s}\right)$, *G*_s_ (*V*_s_), and $r_{f\infty }\left(V_{s}\right)$, $\tau_{\mathrm{rf}}\left(V_{s}\right)$, *G*_f_ (*V*_s_), and then twice the characteristic parameters: *V*_hs_, *k*_s_, *M*_s_, *S*_s_, *A*_s_, *B*_s_, *V*_hf_, *k*_f_, *M*_f_, *S*_f,_ *A*_f_, and *B*_f_.

#### The full‐trace method

The full‐trace method is an iterative algorithm that estimates directly and simultaneously the activation and kinetic functions, and the maximal conductance from all the currents recorded during a voltage‐clamp experiment. Initially, values for the elements of the characteristic curve parameter vector $\left[V_{h},k,M,S,A,B\right]$ are drawn randomly from a prescribed range for each parameter. The algorithm then calculates the values of $r_{\infty }\left(V_{s}\right)$, $\tau_{r}\left(V_{s}\right)$, for each value of *V*_s_, with quations (2) and (3). Next, the set of step currents are reconstructed from Eqn (1). Iteratively, the error between all the modelled current traces and the experimental data traces is minimized with a standard nonlinear least‐squaresd optimization algorithm by adjusting the characteristic curve parameters vector until the error between the modelled and experimental current traces is minimal. The procedure is exactly the same in the presence of two components, now with the extended characteristic curve parameter vector $\left[V_{\mathrm{hs}},\;k_{s},\;M_{s},\;S_{s},\;A_{s},\;B_{s},\;V_{\mathrm{hf}},\;k_{f},\;M_{f},\;S_{f},\;A_{f},\;B_{f}\right]$.

#### Application to simulated data

In order to evaluate the performance of the single‐trace and the full‐trace methods, we simulated an *I*_h_ current that was the sum of two components with the model described in section 3.2 and the parameters shown in Table 1. These parameters were chosen to correspond, as far as possible, to known physiological values in the inner ear (Chen, 1997; Yi *et al*., 2010; Meredith *et al*., 2012).

**Table 1:** Parameters of the two component currents for the evaluation of the single‐trace and full‐trace methods

Component	*V*_h_·(mV)	*k*	*M*·(mV)	*S*	*A*·(ms)	*B*·(ms)	*G*·(nS)
*I*_hs_	−100	−6	−80	80	1000	60	3
*I*_hf_	−130	−9	−80	40	250	40	4

The currents were simulated in response to a voltage protocol with a holding potential of −60 mV and stepped in 10‐mV decrements from −60 to −150 mV, then the post‐step potential was −60 mV. Physiological white noise of 10‐pA amplitude was added.

To determine the best solutions provided by the identification method, we analysed the distribution of the residual error reached from 500 trials. The best solutions that reached successfully the global minima were associated with the first mode of the distribution (28% of the total number of tries), whereas local minima were associated with additional modes clearly spaced from the first mode. To avoid solutions from local minima, we only retained this first mode (smallest 28%) of the total number of tries.

Both identification methods were applied to these simulated data currents for 50 repetitions with random initial parameter values chosen with a uniform distribution inside the search region, which also constrains the minimization function, of size tol = 80% around the simulated current parameter values (Table 1). The search ranges for the single‐trace method are the same for the maximal conductances, between 0 and 1 for the activation gates, and limited to the sum $\left(A+B\right)\pm \text{tol}$, for the time constants.

The individual parameter identification error was estimated using $\varepsilon =\left(p-p_{i}\right)/p\times 100$, where *p*_*i*_ is the identified parameter value and *p* is the simulated current parameter value.

The goodness of fit (GoF) was calculated from the equation: 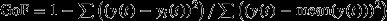, where *y* is the original current trace and *y*_i_ is the identified current trace.

In all experiments, values are presented as mean ± SEM and statistical significance was assessed using Student's *t*‐test at a significance level of 0.05.

### Neural simulation model

A single‐compartment excitable neuron model was used to demonstrate the effects of the two *I*_h_ currents identified in this study. The description of the membrane current flow follows models of cochlear nucleus neurons (Rothman & Manis, 2003a) in the auditory pathway, where maximal conductances are modified to reproduce the firing patterns of sustained and transient vestibular ganglion neurons (Kalluri *et al*., 2010; Yoshimoto *et al*., 2015). We added the *I*_h_ components identified in this study to these models.

(5) $$\begin{array}{c} C\frac{dV}{dt}=-\left(G_{\mathrm{Na}}m^{3}h\left(V-E_{\mathrm{Na}}\right)+G_{\mathrm{KH}}\left(0.85.n^{2}+0.15.p\right)\left(V-E_{K}\right)+G_{\mathrm{KL}}w^{4}z\left(V-E\_K\right)+G_{l}\left(V-E_{l}\right)+I_{\mathrm{hs}}+I_{\mathrm{hf}}\right)+I\left(t\right) \end{array}$$

where *E*_Na_ = 50 mV, *E*_K_ = −77 mV, *E*_h_ = −36 mV (Meredith *et al*., 2012) and *E*_l_ = −67 mV are the reversal potentials. *G*_KH_ = 140 nS, *G*_KL_ = 0 nS for the sustained vestibular ganglion neuron model type, and *G*_KL_ = 50 nS for the transient model type, are the modified maximal conductances; for details see Rothman & Manis (2003a). The values of the *I*_h_ component maximal conductances, *G*_hs_ and *G*_hf_, are identified in this study.

#### Temperature

All the conductances were modelled at physiological temperature, using classical *Q*_10_ values of 3 (Hodgkin & Huxley, 1952).

#### Effect of cAMP

The effect of cAMP, an intracellular cyclic nucleotide that modulates HCN channel activity (Yi *et al*., 2010), on *I*_h_ currents was modelled using data obtained from patch‐clamp recordings of the inner hair cell afferent synapse (Yi *et al*., 2010). Typically, with 200 μm cAMP added to the pipette solution and, additionally, with 200 μm 8‐Br‐cAMP (cAMP analog) added to the external solution, the *I*_h_ current amplitude increases by a factor 1.91, which we utilized to multiply the *I*_h_ model maximal conductances in the case of cAMP presence; the activation curves are shifted by 12 mV toward depolarisation (no effect on the slope), which we modelled by shifting the half‐activations; the fast‐component time constant (probably due to HCN1/HCN2 subunits in the vestibule; Horwitz *et al*., 2010, 2011), like in the cochlea, is reduced by a factor 2.87, and the slow‐component kinetic is reduced by a factor 5.31, which values we used to divide the magnitude of the respective component kinetic functions).

#### Implementation

All the simulations and algorithms of this study were implemented in Python. The current‐trace errors were calculated as the sum‐of‐squares difference between the model and experimental traces at all recorded time points. Parameter identification to minimise this error was done with the *fmin_powell* function of the *scipy.optimize* Python package.

## Results

### Discrimination of the different components in simulated data

Both parameter identification methods were applied to simulated *I*_h_ currents, as described in the methods. Figure 1A, B, E and F shows the individual best trial results (grey lines) and the averages (black bold lines), which are the final results for both methods. The single‐trace method individual trial activation function identifications (Fig. 1A) are confounded and do not permit, when averaged, to distinguish the activations of the two current components. The kinetic components are well separated for the two methods (Fig. 1B and F) but for the single‐trace method they are not a close fit to the kinetics used to generate the simulated data (Fig. 1B), unlike with the full‐trace method (Fig. 1F). Figure 1C and D shows the simulated (grey traces) and reconstructed (black traces) currents (without deactivation currents for clarity) with parameters identified with the single‐trace method (Fig. 1A and B). Figure 1C shows the quality of reconstruction when the two components are together and Fig. 1D when the two components are separated. The single‐trace method permits a very good reconstruction of the combined currents (GoF = 0.99) but shows a poor reconstruction for the separated currents (fast‐component GoF = 0.82, slow‐component GoF = 0.91) due to the misidentification of the activation curves (Fig. 1A).

The full‐trace method individual trial activation identifications (Fig. 1E) are well separated and permit, when averaged, to distinguish the two components of activation. The full‐trace method shows a very good reconstruction of the combined currents (GoF = 0.99; Fig. 1G) as well as the separated currents (fast component GoF = 0.99, slow component GoF = 0.99; Fig. 1H).

Table 2 relates the relative error between each simulated and identified parameter by the single‐trace and full‐trace methods. The averaged error per parameter value for the single‐trace method is 23.4%. In addition, it is impossible, between the slow and fast components, to discriminate their half‐activations, V_hs_ and V_hf_ (*P *= 0.13), their widths, *S*_s_ and *S*_f_, or their maximal conductances, *G*_s_ and *G*_f_. The averaged error for the full‐trace method is 4.1%. This method is particularly better than the single‐trace method on activation (compare Fig. 1A with Fig. 1E) but also on kinetic identification (the single‐trace kinetic amplitude errors are largely > 20% against < 10% for the full‐trace method; compare Fig. 1B and F). Most of the parameter identification errors for the full‐trace method are < 5%, and a few are < 1%. Furthermore, statistically significant differences between the two *I*_h_ components are found in all relevant cases.

**Table 2:** Relative parameter identification evaluation for single and full‐trace methods

Method	*V*_h_	*K*	*M*	*S*	*A*	*B*	*G*
Single trace							
Slow	16 ± 7.1	30 ± 24.0	21 ± 10.1	36 ± 7.9	18 ± 14.5	28 ± 28.6	23 ± 5.8
Fast	7 ± 4.0	34 ± 17.7	22 ± 9.0	20 ± 23.7	26 ± 15.5	35 ± 17.4	12 ± 7.0
*P*	0.13	< 0.01	0.7	0.31	< 0.01	< 0.05	< 0.01
Full trace							
Slow	0 ± 0.1	4 ± 6.3	2 ± 21.2	7 ± 20.6	10 ± 15.7	16 ± 29.2	3 ± 5.3
Fast	0 ± 0.5	0 ± 5.2	0 ± 14.2	2 ± 20.5	9 ± 27.6	5 ± 20.6	0 ± 8.3
*P*	< 0.01	< 0.01	0.57	< 0.01	< 0.01	< 0.01	< 0.01

The first two lines for both methods show the errors in percentage, compared to the original parameters. The mean single‐trace method error per parameter value is 23.4%. The mean full‐trace method error is 4.14%. The third line indicates the significance of difference (*P*‐value). The single‐trace method does not find significant differences between half‐activations and the Gaussian width of kinetics. When relevant, the differences are always significant for the full‐trace method.

**Figure 2:**
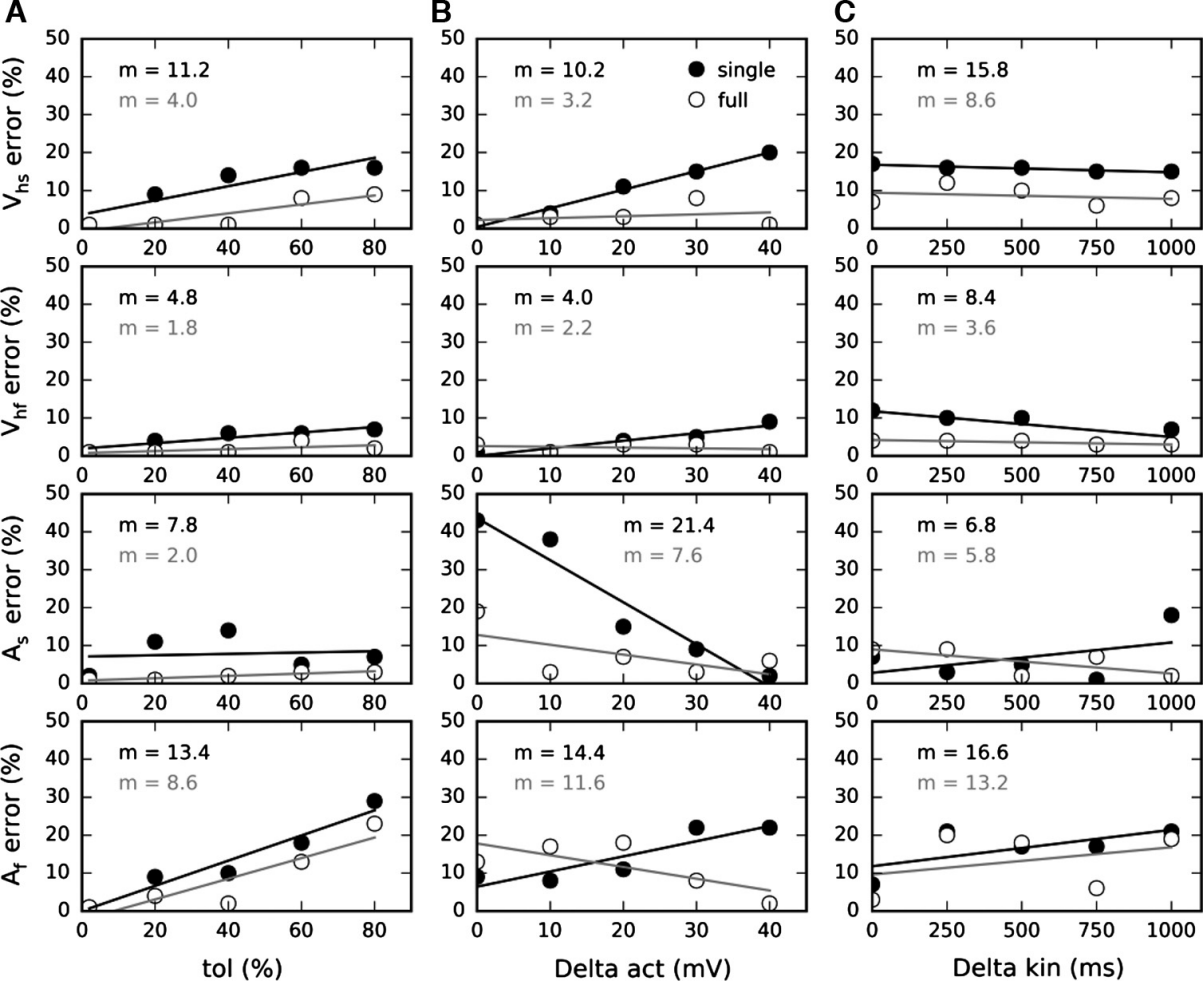
Sensitivity analysis. The half‐activations, M, and the kinetics amplitudes, A, errors (% of the genuine parameters) are shown as a function of (A) tolerance (tol), (B) difference between half‐activations (delta act) and (C) difference between kinetics amplitude (delta kin). The solid and open points correspond respectively to the single‐ and full‐trace identification methods. The lines show the tendencies. The single‐trace method results in averaged errors (m) that are always larger than the full‐trace method, whatever the tested parameter**.**

To test the sensitivity of the two identification methods, tests were made with either (i) different sizes of the initial search zone (tolerance), (ii) different distances between the half‐activations, V_h_, of the two current components (by varying *V*_hs_ from −130 to −90 mV), or (iii) different distances between the kinetic amplitudes (by varying *A*_s_ from 250 to 1250 ms). Figure 2 shows this analysis for the two methods. Identification errors of the principle parameters (*V*_hs_, *V*_hf_, *A*_s_, and *A*_f_) are shown as a function of the size of the search zone (*tol*), the difference between half‐activations (Delta act: ¦*V*_hs_−*V*_hf_¦), or the difference between the kinetics amplitudes (Delta kin: ¦*A*_s_−*A*_f_¦). Unsurprisingly, for all the shown parameters the identification becomes more and more difficult when the search zone increases, but more substantially for the single‐trace method (Fig. 2A). Variation of the difference between half‐activations does not affect the half‐activation parameter identification by the full‐trace method but increases the error of the single‐trace method (Fig. 2B). The fast and slow kinetics amplitudes are mostly more easily identified by both methods when the difference between the half‐activations is large. Increasing the difference between kinetics amplitudes results in slightly better identification of the half‐activations by both methods and, for the full‐trace method, identification of the slow kinetic amplitude, contrary to the fast kinetic amplitude (Fig. 2C). The averaged error values (*m*) are always smaller for the full‐trace method compared to the single‐trace method.

### Application to recorded *I*_h_ currents

**Figure 3:**
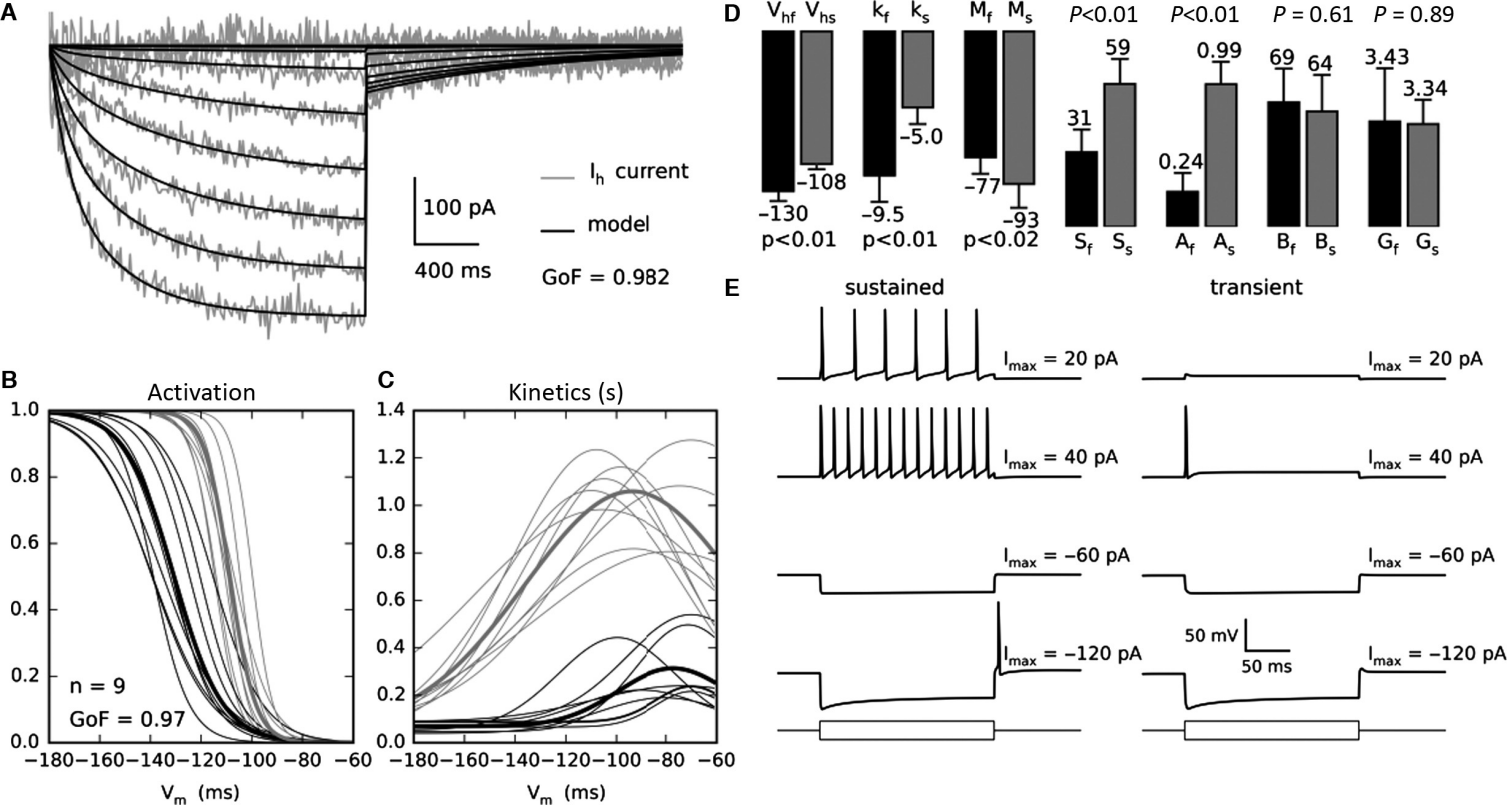
The two‐component *I*_h_ current parameters identification. (A) Recorded *I*_h_ currents (grey trace) in response to a voltage‐clamp protocol with a holding potential of −60 mV and a step potential from −60 to −150 mV decreased in steps of 10 mV. Reconstruction by the model (black trace). (B) Half‐activation of the slow (grey) and fast (black) activated components are respectively at −108.6 and −130.6 mV, the slopes are respectively of −5.0 and of −9.5. Thin lines are best fits for each of the nine cells (average of 14 best tries from 50); thick lines are average fit over the nine cells. (C) The kinetic amplitudes of the slow‐ and fast‐activated components are respectively 0.99 and 0.24 s and the widths (parameters S) are, respectively, 59.8 and 31.2. (D) Parameter values and SDs are shown for the slow (grey bars) and fast (black bars) components. (E) Firing patterns and hyperpolarisation sags of sustained and transient vestibular neurons models, provided with both *I*_h_ components, in response to −120, −60, 20 and 40 pA current‐clamp stimulation. The models reproduce actual data (Kalluri *et al*., 2010; Yoshimoto *et al*., 2015).

We applied the full‐trace method on *I*_h_ current recordings from mouse vestibular neurons (*n *= 9) assumed to be composed of at least two activated components given the two exponentials needed for single‐trace fitting (Meredith *et al*., 2012), and the presence of several HCN subunits, including in the vestibular periphery (Horwitz *et al*., 2010, 2011), not isolated pharmacologically in the cochlea (Chen, 1997; Yi *et al*., 2010). Figure 3 shows the application of the full‐trace method to the recorded *I*_h_ currents, the parameters identified, and the simulations of the sustained and transient vestibular neuron models provided by the newly identified *I*_h_ component models. Figure 3A shows an example of the *I*_h_ current (grey trace) in response to a voltage‐clamp protocol with a holding potential of −60 mV and a step potential from −60 to −160 mV stepped in decrements of 10 mV. The black trace shows the reconstruction of these currents after identification. For each recorded cell, the fitting of the experimental traces involves the averaging of the 14 best repetitions from 50 trials, resulting in a GoFof 0.98 in the example shown in Fig. 3A and an average GoF of 0.97 over the nine recordings. In Fig. 3, panels B and C show, respectively, the activation and kinetic curves obtained for all the *I*_h_ recordings (*n *= 9). The thin lines represent the averaging of the 14 best trials on one particular cell (the final result of the algorithm applied to one particular cell, or the equivalent of the bold line in the Fig. 1E). The bold lines represent the averaging of the results obtained for the nine cells. Figure 3D shows the identified parameter values, which indicate a fast component that is a low‐voltage‐activated current (*V*_hf_ = −130.6 mV) and slow component that is a high voltage activated current (*V*_hs_ = −108.6 mV), with significantly different activation slopes *k*_f_ and *k*_s_ of, respectively, −5.1 and −9.6. The principle kinetic characteristics are also significantly different, with fast and slow maximum time constants of, respectively, 0.244 and 0.995 s, and widths *S*_f_ and *S*_s_ of, respectively, 59.8 and 31.2. The pairwise parameters of the two components are significantly different in all cases apart from the kinetic bases (*B*_f_ and *B*_s_) and the maximal conductances (*G*_f_ and *G*_s_).

Figure 3E shows the responses of the vestibular ganglion neuron models (sustained, left panel and transient, right panel; see Materials and methods) provided by both identified *I*_h_ components, evoked by positive and negative current‐clamp steps. The firing patterns differentiating the two basic categories of vestibular ganglion neurons (Kalluri *et al*., 2010; Yoshimoto *et al*., 2015) are well reproduced by the models (left and right upper panels). The sag which is known to reflect the presence of the *I*_h_ currents in vestibular ganglion neurons (Chabbert *et al*., 2001b), in response to hyperpolarising current steps, is reproduced by the model in both neuron model types (left and right lower panels).

To confirm that the two‐component model is indeed the most parsimonious fit to the data, we applied the full‐trace method with only one current component and the GoF was 0.93 (significantly different than with two components, *P* < 0.02). The GoF obtained with a three‐component current model is slightly better but not significantly different from the GoF obtained with the two‐component model; thus if the actual *I*_h_ currents are composed of more than two components, their effect is negligible.

### *I*_h_ components shape action potential excitability and EPSPs

The *I*_h_ currents are known, in the inner ear, to shape EPSPs and modulate excitability in neurons (Yi *et al*., 2010). To see whether the two components contribute differently to shaping neuronal excitability and EPSPs, we have simulated the sustained and transient neuron models with both *I*_h_ components and with each isolated component, to evaluate AP latencies due to the *I*_h_ currents and, in the case of *G*_Na_ = 0 nS (modelling the presence of TTX), to see the influence they have on EPSP shape.

**Figure 4:**
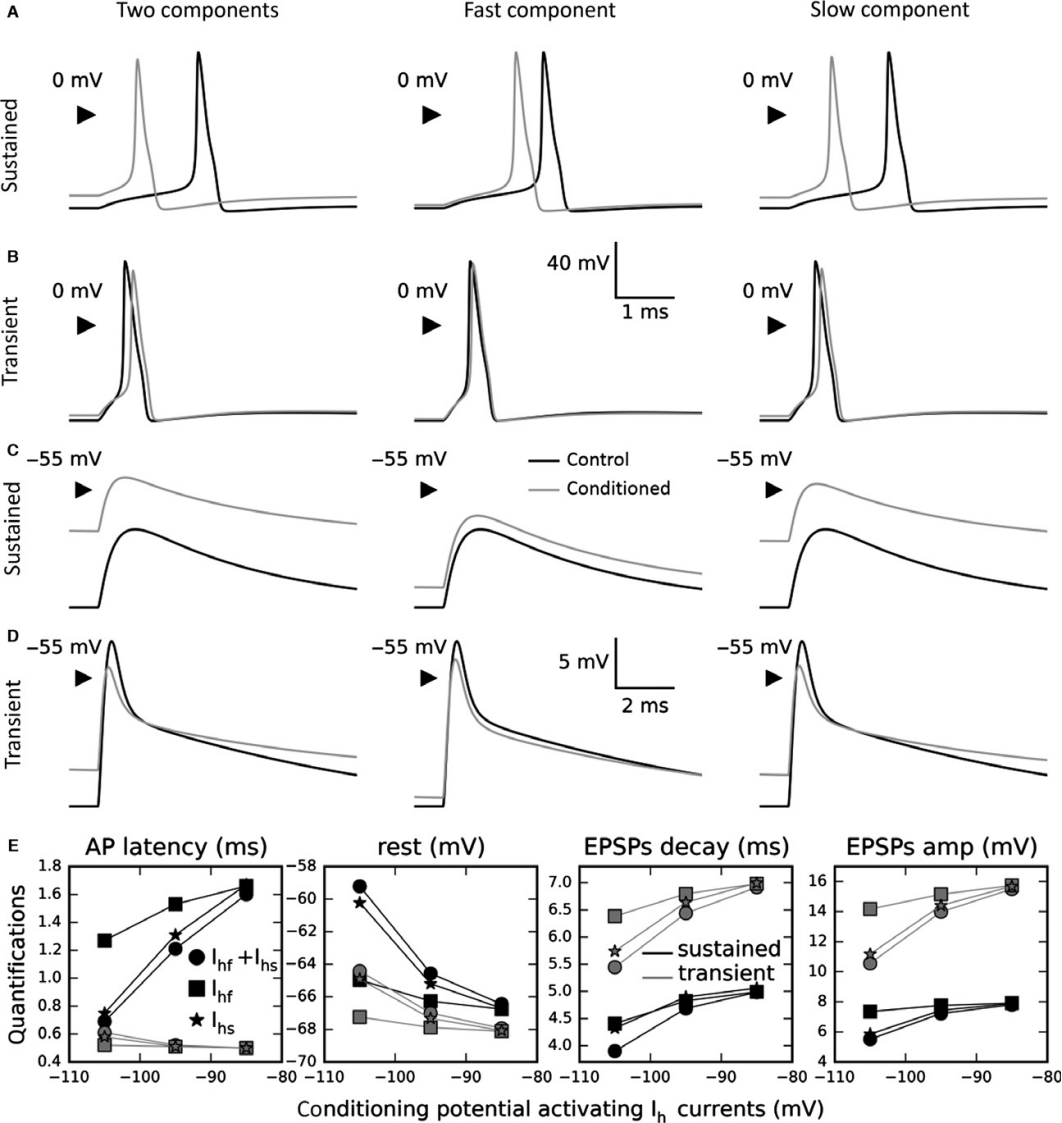
AP excitability and EPSP shaping in control case and after *I*_h_ conditioning at −105 mV, for fast and slow components, on sustained and transient vestibular neuron models. (A) APs firing in sustained neuron in control case (black lines), and with both *I*_h_ components (left panel, grey line), fast component (middle panel, grey line), and slow component (right panel, grey line). (B) Same simulation with transient neuron model. (C) Same protocol with $G_{Na}=0nS$ (modelling effect of TTX) showing the EPSP shaping by different *I*_h_ components on sustained neurons. (D) Same protocol on transient neuron. (E) Quantifications for different conditioning hyperpolarizing potentials of the AP latency, resting potential, EPSP exponential decay time constant and EPSP amplitude, in sustained neurons (black traces) and in transient neurons (grey traces). The circle curves represent the simulations with both components, the square curves the simulation with the fast component only, and the star curves the simulation with the slow component only. The results show that all these variations are principally due to the slow component on both sustained and transient neuron models.

Figure 4A shows APs fired in response to a 10%‐above‐threshold double‐exponential current stimulation (rise time constant 0.05 ms, decay time constant 5 ms; Sadeghi *et al*., 2014). The black trace is the control case, without *I*_h_ currents in the model, and the grey traces are the response of the sustained neuron, after a conditioning protocol activating the *I*_h_ current at −105 mV (a 500‐ms hyperpolarisation, which is long enough to reach a steady‐state value for the *I*_h_ currents but not large enough to induce spontaneous AP firing, followed by a delay of 5 ms at rest before inducing the AP). Left central, and right panels show the response of the model provided with both *I*_h_ components, fast component only, and slow component only, respectively. The results show that the latencies of neuronal firing are reduced, mainly driven by the slow component. Figure 4B shows the simulations done with the transient neuron model. Now the *I*_h_ currents introduce a very small increase in the latency.

Figure 4C and D shows the same simulations but without the sodium conductance, to evaluate the influence of both *I*_h_ components on the shape of the EPSP, with the sustained and transient neuron models, respectively. Changes in the time course or amplitude of the EPSP could influence the temporal requirements for transmitter release to generate an AP. Once again, the slow component contributes more than the fast component in affecting the shape of the EPSPs, resulting in a smaller amplitude and faster time course. In addition, the simulations show that the EPSP shape and the resting membrane potential are more influenced by the *I*_h_ currents in the sustained neuron than in the transient neuron model.

Quantification of these effects for several conditioning potentials (Fig. 4E) shows that in sustained neurons (black traces), as a function of the conditioning potential, the AP latencies (measured from the stimulation to the peak) vary from 0.65 to 1.6 ms, the resting potentials decrease from −59 to −66 mV, the EPSP exponential decays vary from 3.9 to 5.0 ms, compatible with data (Meredith *et al*., 2012; Sadeghi *et al*., 2014), and the EPSP amplitudes increase slightly from 5.5 to 7.5 mV. In transient neurons (grey traces), as a function of the conditioning potential, the AP latencies vary from 0.65 to 0.50 ms, the resting potentials decrease from −64.5 to −68 mV, the EPSP exponential decays vary from 5.4 to 6.8 ms, and the EPSP amplitudes increase significantly from 10.5 to 15.5 mV. All these variations are principally due to the slow component in both sustained and transient neuron models.

### Neural simulations with identified *I*_h_ currents

**Figure 5:**
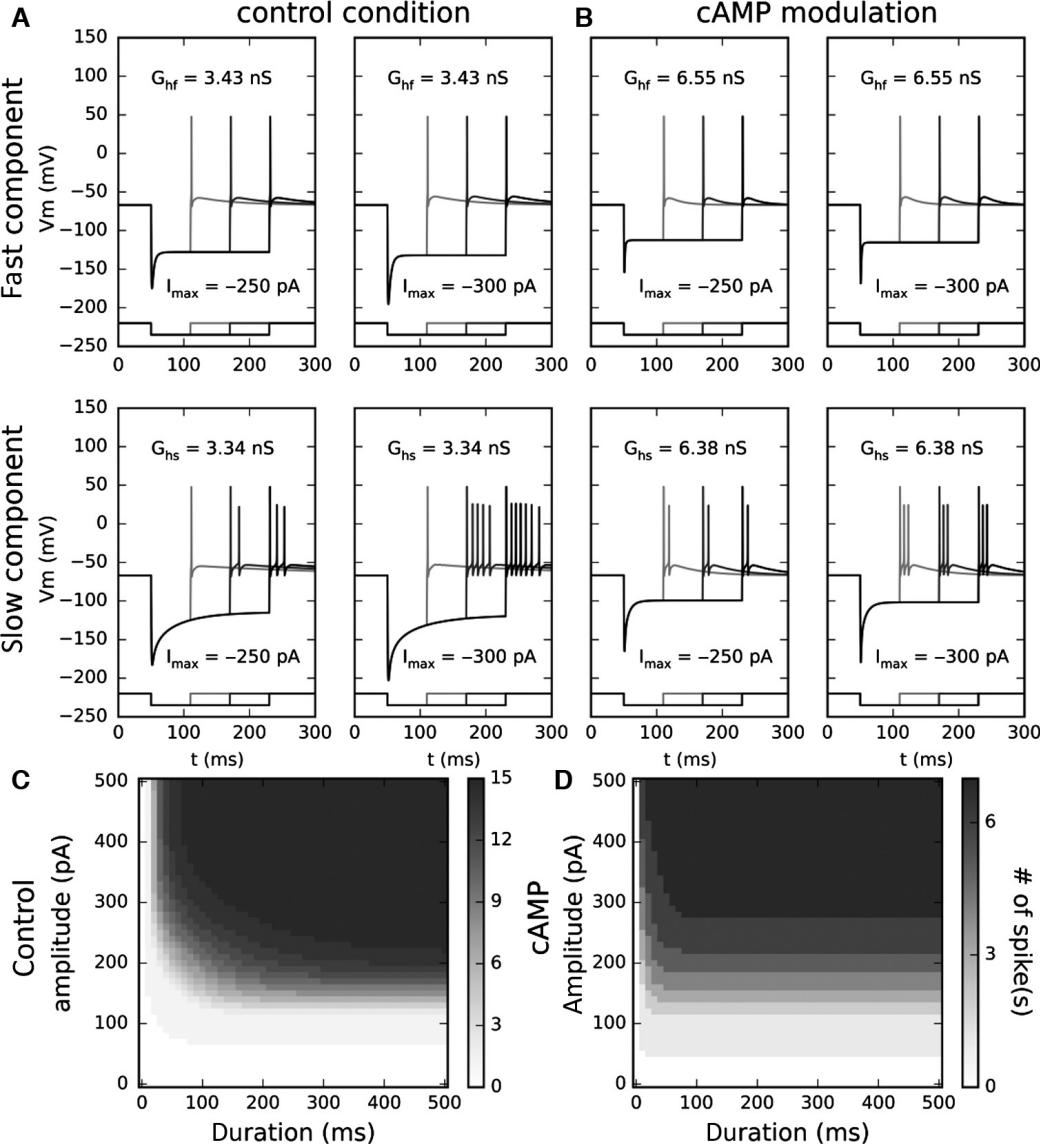
Neural response (AP number) as a function of amplitude and duration in control conditions and in the presence of cAMP. (A) AP firing induced by slow and fast *I*_h_ in response to inhibitory stimulations. Upper panels, in response to −250‐ or −300‐pA steps, the neuron model with fast component only triggers only one AP even for long stimulations. Left lower panel, in response to a −250‐pA step, with slow component only, the model triggers one, two or three APs as a function of the stimulation duration. Right lower panel, in response to a −300‐pA step, the model triggers one, five or seven APs as a function of the stimulation duration. (B) AP firing induced by slow and fast *I*_h_ modulated by cAMP, in response to inhibitory stimulations. Upper panels, in response to −250‐ or −300‐pA steps, the neuron model with only the fast component triggers only one AP even for long stimulations. Lower panels, in response to −250‐ and −300‐pA steps, with only the slow component, the model triggers respectively two and three APs whatever the stimulation duration. (C) With the slow component only, in control conditions, the neural response is dependent on both duration and amplitude and the neural response codes for the duration (relative amplitude independence) for a stimulus > 350 pA, and for the amplitude (relative duration independence) for a stimulus > 200 ms. (D) Now in the presence of cAMP, the neural response becomes totally independent of the duration for a stimulus > 50 ms and the neural response codes exclusively for the stimulus amplitude.

To further highlight the effect of each component (Fig. 5), we added only one of the two *I*_h_ components at a time to the sustained‐neuron model. The model was stimulated with either a moderate negative hyperpolarising current, A = −250 pA (Fig. 5A, left panels) or a slightly stronger current, A = −300 pA (Fig. 5A, right panels), with varying durations. Whatever the stimulation amplitudes or durations, the fast component is able to fire only one AP (Fig. 5A, upper panels). The slow component induces bursts of one to seven APs as a function of the stimulation duration and this effect increases with stimulation strength (Fig. 5A, lower panels). The results with both components present (not shown) are very similar to those with the slow component only.

In the same conditions of stimulation, we simulated the firing of the cells with the modelled effect of cAMP (Fig. 5B), which increases current amplitude, shifts the activation curves and reduces the two kinetic components differently (see Materials and methods). Whatever the stimulation amplitudes and durations (Fig. 5B, upper panels) the fast component still is able to fire only a single AP. Interestingly, the slow component induces either bursts of two or three APs as a function of the stimulation strength, now independently of the stimulation duration (Fig. 5B, lower panels).

To better understand the role of the slow component, which is clearly involved in shaping the stimulation coding, we have fully tested the neural response (number of APs fired after inhibitory stimulation) of this model as a function of the stimulation duration and amplitude, in control conditions (Fig. 5C) and in the presence of cAMP (Fig. 5D). In control conditions the neural response is not clearly correlated with one or the other variable. The neuron seems to code for the duration of stimuli > 350 pA, and for the amplitude of stimuli > 200 ms. However, in the presence of cAMP the neural response becomes totally independent of the duration for stimuli > 50 ms and the neuron codes then exclusively for the stimulus amplitude.

In addition, we tested the transient vestibular neuron model with the same protocols, but the large amplitude of a low‐voltage‐activated potassium current, probably composed of Kv1 and KCNQ channels, in this cell, precluded rebound firing of more than one AP, as already shown (Kalluri *et al*., 2010; Yoshimoto *et al*., 2015) whatever the activated *I*_h_ component.

## Discussion

### Hyperpolarisation‐activated currents

This study shows that the *I*_h_ current in vestibular ganglion neurons is composed of a slow and a fast component which are activated at different voltages, quite similar to the *I*_h_ current in the guinea pig spiral ganglion (Chen, 1997), the rat primary auditory afferent dendrites (Yi *et al*., 2010) and the vestibular calyx afferent of Mongolian gerbils (Meredith *et al*., 2012). This is the first identification and modelling of separate *I*_h_ components.

Suprathreshold EPSPs (slightly larger than spontaneous EPSPs from data) that generated an AP were modelled to measure the effect of the *I*_h_ current components on ganglion cell firing properties. We have also modelled EPSPs, in the absence of cell firing, to provide information about the effect of the two *I*_h_ current components on the response to synaptic input from hair cells to afferent terminals (independently of their effect on firing). These *I*_h_ currents influence both AP firing and EPSP shape. In sustained neurons, the AP latency could be strongly decreased by the activation of the *I*_h_ current, due to depolarisation of the resting potential, and the EPSP time course also is decreased, sharpening the temporal response to transmitter release. Both of these effects could allow the regulation of firing rate in vestibular neurons, as well as in the cochlea (Yi *et al*., 2010). In transient neurons, surprisingly, the AP latency very slightly increases, but the resting potential, decay time and amplitude vary similarly to the sustained neurons. Interestingly, the presence of low‐voltage‐activated potassium conductance in these neurons results in EPSPs with a double exponential decay (Fig. 4D), like the calyx EPSCs (Sadeghi *et al*., 2014). For all these conclusions, the main effect is due to the slow *I*_h_ component.

Further computer simulations indicated that these two *I*_h_ components influence the firing behaviour of these cells in response to inhibitory (hyperpolarising) stimulation of sufficient magnitude and duration to generate rebound APs. The components allow the coding of different characteristics of the stimulation. The fast component permits the firing of a single AP at the end of the stimulation, regardless of stimulation duration or amplitude, whereas the slow component rather permits coding of the stimulation duration or amplitude by the number of rebound APs fired (and this is further modified in the presence of cAMP; see below). These results are valid only for the sustained vestibular ganglion neurons. The high sensitivity of the rebound AP to the low‐voltage‐activated potassium current (Kv1 and KCNQ; Hurley *et al*., 2006), means the transient neurons do not fire more than one AP, even for high stimulation duration and amplitude.

The stimulation potentials used in the computer simulations underpinning the conclusions of this study, in particular concerning the firing of rebound APs, may seem surprisingly long and hyperpolarised, but the spontaneous APs recorded in the vestibular calyx terminal (transient neurons) are followed by a strong hyperpolarisation reaching −160 mV with duration of ∼100 ms (Meredith *et al*., 2012). This form of hyperpolarisation drives the electrophysiological behaviour presented in this study. However, it would be worth knowing whether this strong hyperpolarisation is also present in the sustained neurons, which are more likely to fire rebound APs. Such hyperpolarisations have not yet been seen in these neurons and this could confirm the eventual functional role of the *I*_h_ currents in these neurons. That said, the sustained neurons do not need to give information about the stimulus duration because of their regularity, but this functionality could be interesting to code the strength of the preceding hyperpolarisation.

*I*_h_ currents are thought to contribute to setting the resting membrane potential (He *et al*., 2014). This is the case for the slow component, because of its ‘high’ voltage activation (closer to the resting membrane potential) but still only after conditioning induced by preceding strong hyperpolarisation; however, it is more difficult for the fast component because of the very low voltage activation (Fig. 3B and C). So, if the slow component could be expected to depolarise the membrane resting potential, the fast component could be only expected to regulate the firing of the neurons, for example by inducing rebound APs.

The most popular hypothesis concerning the presence of two components in the hyperpolarisation‐activated cationic current is that the different electrophysiological behaviours arise from channel populations consisting of different mixtures of HCN subunits (Liu *et al*., 2014). Concerning the mouse vestibular ganglion, here we show the presence, at room temperature, of a fast activated component (200–400 ms) and a slow activated component (800–1200 ms) that could be principally induced respectively by HCN1/HCN2 and HCN4 subunits, knowing their kinetics (Santoro *et al*., 2000), and this would be compatible with previous results in the vestibular periphery (Horwitz *et al*., 2010, 2011).

A study in the superior paraolivary nucleus showed that an *I*_h_ current has to be associated with a transient calcium current for a neuron to be able to burst in response to inhibitory current stimulation (Kopp‐Scheinpflug *et al*., 2011). Our study shows, without neuromodulation, that the fast component effectively provides only one AP even for strong or long inhibitory stimulation, but in these conditions the slow component can stimulate bursting responses (without any other association) where the number of APs is dependent on the stimulation amplitude or duration. A next step in the study of ionic channel associations could be this calcium effect with each of the *I*_h_ components, and the specific HCN subunits with which it is required to allow an inhibitory current to cause stimulation bursting.

### cAMP modulation

In the inner ear, cAMP has been shown to strongly reduce the time constants and to shift the activation curves towards depolarisation differently according to the related *I*_h_ current component (Yi *et al*., 2010). With our cell model, we have shown that the presence of cAMP does little to change the effect of the fast component. However, the slow component loses its ability to signal stimulus duration and amplitude and becomes only a clear stimulus amplitude indicator. Again, these results are valid only for the sustained‐type neurons, and transient‐type neurons do not fire more than one AP, even for high stimulation duration or amplitude. It has also been show in this study that the cAMP modulation does not affect the *I*_h_ influence on AP excitability nor the shape of EPSPs.

It has been proposed that the *I*_h_ conductance regulates the firing activity or resting membrane potential in the inner ear (Chen, 1997; Liu *et al*., 2014). However, we found that the half‐activation potentials of both components are too hyperpolarised to be substantially associated with these. This discrepancy could be due to the young age of the tested mice. To go further, it was shown hyperpolarization‐activated cation currents are shifted at adult ages by ~30 mV toward depolarisation (Khurana *et al*., 2012). It could be interesting to apply the algorithm at different stages of development to see at which moment the *I*_h_ currents are able to regulate firing rate and, in this regard, be effectively modulated by cAMP.

Seven different neurotransmitters are known to be released by the lateral efference in the cochlea and vestibule (Ryugo *et al*., 2011). Their effect on cAMP and further on *I*_h_ currents could be the way by which lateral efference in the cochlea or brainstem vestibular nuclei modulates the firing pattern or the resting potential of the afferent pathways. In fact, efferent transmitters could potentially influence *I*_h_ gating by modulating intracellular levels of cyclic nucleotides. HCN channels can be potentiated by direct binding of cAMP through the CNBD, and intracellular cAMP levels could change via G‐protein‐coupled inhibition or activation of adenylyl cyclase (Meredith *et al*., 2012). It was speculated that elevating levels of intracellular cyclic nucleotides could enhance firing in calyx afferents through *I*_h_ (Meredith *et al*., 2012). In our study, however, given the very low potentials at which they are activated, it is not likely the *I*_h_ current would be able to influence directly the vestibular afferent's firing, and it would more probably affect the rebound APs, AP excitability or EPSP shaping after hyperpolarisation, and this would be principally driven by the slow component. Once again, this is valuable at P5–P8, and the activation shifting at adult ages could strongly modulate this assumption.

A future study could model the different neurotransmitters involved in the regulation of the neural afferent activities in the inner ear and evaluate their impact associated with the presence of different HCN channels on the afferent pathway. Also, it would be interesting to know whether and what kind of neuromodulation would be responsible for the switching of the slow component to be a specific stimulus duration indicator, as an antagonist effect of the switching to a specific stimulus amplitude indicator, as with cAMP.

### Algorithm

Several studies have revisited the full‐trace method: using data from patch‐clamp experiments to identify the parameters of Markov models on rat cortex pyramidal neurons (Gurkiewicz & Korngreen, 2007); to highlight the neural dynamics in lobster lateral pyloric neurons (Nowotny *et al*., 2008); and to estimate a conductance gradient along a dendrite (Keren *et al*., 2009). Only one study of this type concerns the identification of two ionic populations, one inactivated and one non‐inactivated potassium conductance (Vavoulis *et al*., 2010). This context is more favourable than the one of our study, indeed the presence of one inactivated conductance implies easier identification of the non‐inactivated conductance once the first is inactivated. Furthermore, this study does not give any details of the protocols or comparison with a single‐trace method. Our study shows that the full‐trace method also identifies the conductance parameters in the new context of two non‐inactivated voltage‐dependent ionic currents, whatever the characteristics of the two components, much more accurately than the single‐trace method based on the fitting procedure of Hodgkin & Huxley (1952), from voltage‐clamp data.

This study is focused on *I*_h_ currents but would be appropriate for other neurons or sensory cells where different ionic currents are involved that may not be able to be perfectly pharmacologically isolated. In particular, there are two potassium conductances (low‐voltage‐activated and high‐voltage‐activated) all along the auditory pathway (Santos‐Sacchi, 1993; Mo *et al*., 2002; Szabó *et al*., 2002; Rothman & Manis, 2003a; Rusznák & Szucs, 2009). The full‐trace method could be used to verify the good identification of the activations and kinetics of these potassium conductances, and to evaluate the selectivity of the pharmacological blockers.

Moreover, neuronal activity depends sometimes on more than seven voltage‐dependent ionic currents, for example, in the developing inner hair cell AP firing (Marcotti, 2012). Pharmacological tools do not necessarily exist for all these channels. Further, even if the pharmacological tools do exist, because of the sigmoidal shaped dose–response curve, the inhibition of a current is never total, even with very strong blocker concentration. Thus, the ability of the full‐trace method to identify conductance parameters was shown here in the presence of two non‐inactivating ionic components but could be explored in tougher conditions. A study has shown the full‐trace method could be applied with one inactivating and one non‐inactivating ion channel population (Vavoulis *et al*., 2010). A novel study could examine the full‐trace method's ability to identify two non‐inactivating and one inactivating population, enzymatically separated, such as in the spiral ganglion neurons (Szabó *et al*., 2002).

We have tested the algorithm on patch‐clamp data that does not include a separate de‐activation protocol, apart from a return to the pre‐step potential following the activation step. To overcome this lack, we used a symmetric kinetic model. In order to improve knowledge of the *I*_h_ current (for depolarised potentials) it would be interesting to use this algorithm on data including a de‐activation protocol with an asymmetrical kinetic model.
